# BCM: toolkit for Bayesian analysis of Computational Models using samplers

**DOI:** 10.1186/s12918-016-0339-3

**Published:** 2016-10-21

**Authors:** Bram Thijssen, Tjeerd M. H. Dijkstra, Tom Heskes, Lodewyk F. A. Wessels

**Affiliations:** 1 Computational Cancer Biology, The Netherlands Cancer Institute, Plesmanlaan 121, 1066 CX Amsterdam, The Netherlands; 2 Max Planck Institute for Developmental Biology, Spemannstrasse 35, 72076 Tübingen, Germany; 3 Centre for Integrative Neuroscience, University Clinic Tübingen, Otfried-Müller-Strasse 25, 72076 Tübingen, Germany; 4 Radboud University Nijmegen, Institute for Computing and Information Sciences, Heyendaalseweg 135, 6525 AJ Nijmegen, The Netherlands; 5 Faculty of EEMCS, Delft University of Technology, Mekelweg 4, 2628CD Delft, The Netherlands

**Keywords:** Bayesian statistics, Sampling, Markov chain Monte Carlo, Sequential Monte Carlo, Nested sampling

## Abstract

**Background:**

Computational models in biology are characterized by a large degree of uncertainty. This uncertainty can be analyzed with Bayesian statistics, however, the sampling algorithms that are frequently used for calculating Bayesian statistical estimates are computationally demanding, and each algorithm has unique advantages and disadvantages. It is typically unclear, before starting an analysis, which algorithm will perform well on a given computational model.

**Results:**

We present BCM, a toolkit for the Bayesian analysis of Computational Models using samplers. It provides efficient, multithreaded implementations of eleven algorithms for sampling from posterior probability distributions and for calculating marginal likelihoods. BCM includes tools to simplify the process of model specification and scripts for visualizing the results. The flexible architecture allows it to be used on diverse types of biological computational models. In an example inference task using a model of the cell cycle based on ordinary differential equations, BCM is significantly more efficient than existing software packages, allowing more challenging inference problems to be solved.

**Conclusions:**

BCM represents an efficient one-stop-shop for computational modelers wishing to use sampler-based Bayesian statistics.

**Electronic supplementary material:**

The online version of this article (doi:10.1186/s12918-016-0339-3) contains supplementary material, which is available to authorized users.

## Background

There is an increasing interest in using Bayesian statistics for the analysis of computational models in biology [1–4]. With Bayesian statistics, the unknown parameters of a computational model are assigned a probability distribution describing their uncertainty. This distribution can be updated from prior information to give the posterior probability distribution, using Bayes’ theorem:

1 $$ P\left(\theta \Big|X,\mathrm{\mathcal{M}}\right)=\frac{P\left(X\Big|\theta, \mathrm{\mathcal{M}}\right)P\left(\theta \Big|\mathrm{\mathcal{M}}\right)}{P\left(X\Big|\mathrm{\mathcal{M}}\right)} $$

where *Θ* represents the parameters, *X* the measurement data and ℳ the computational model. Furthermore, the marginal likelihood, or evidence, can be used to discriminate between different computational models. It can be calculated by marginalizing the parameters:

2 $$ P\left(X\Big|\mathrm{\mathcal{M}}\right)={\displaystyle \int P\left(X\Big|\theta, \mathrm{\mathcal{M}}\right)P\left(\theta \Big|\mathrm{\mathcal{M}}\right)d\theta } $$

Typically, neither the posterior probability nor the marginal likelihood can be calculated directly, but sampling algorithms can be used to estimate them [5–16]. These sampling algorithms are computationally demanding, especially when the number of parameters is large and when the computational model is expensive to simulate. Typical models in systems biology indeed carry many parameters and are expensive to simulate [17]. Additionally, the posterior probability distributions arising from such models are usually complex, containing multiple modes and ridges that are difficult to traverse [18]. Bayesian analysis of such systems biology models thus requires the use of advanced sampling algorithms. Since these sampling algorithms each have unique characteristics and can be more or less suitable for a particular task, it would be beneficial to have various algorithm easily available.

BCM, a toolkit for the Bayesian analysis of Computational Models using samplers, provides efficient, multithreaded implementations of eleven algorithms for calculating posterior probabilities and marginal likelihoods.

The BCM toolkit focuses on computational models that involve simulations or extensive calculations. Examples of such computational models are systems of ordinary differential equations describing biochemical reactions; or steady-state signaling networks, where the activity levels may be calculated in diverse ways. These computational models are in contrast to statistical models that can be specified in the BUGS or Stan languages. For such statistical models, excellent software packages already exist [19, 20]. For the computational models that are targeted by BCM, several alternative software packages also exist [5, 21–23]. However, each of these packages implements only a single type of sampling algorithm and most of them focus on one particular type of computational model. In contrast, with BCM the user can choose from eleven sampling algorithms and the plugin architecture allows diverse types of models. Thus, BCM represents a one-stop-shop for Bayesian analysis of systems biology models, where the user has a high chance of finding a suitable algorithm for the analysis of the user-defined model.

## Implementation

**Fig. 1:**
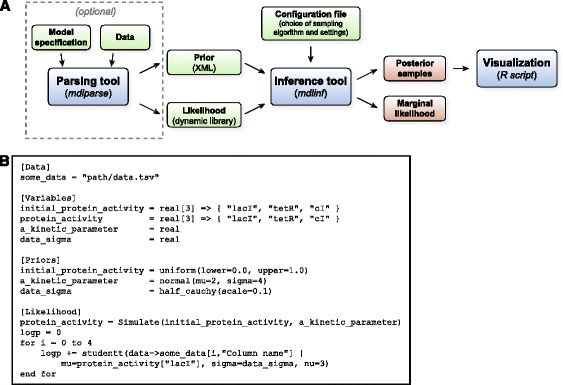
Overview of BCM. **a** The inference tool is the main component of BCM, providing three classes of algorithms for generating samples from posterior probability distributions and calculating estimates of the marginal likelihood. The parsing tool can optionally be used to generate the prior and likelihood files from a model description file and data. **b** Excerpt of a model description file. The model parsing tool can parse this file, load the relevant data, and output C++ source code for a dynamic library that evaluates the likelihood function. In this example, the “Simulate()” function still has to be implemented by the user with a desired simulation method

BCM consists of three components: an inference tool, a model parsing tool and an R script for further analysis and visualization (see Fig. 1a).

The inference tool (*mdlinf*) is the main component of BCM. It uses a specified sampling algorithm to generate samples from the posterior probability distribution and to calculate a marginal likelihood estimate. Error bounds for the marginal likelihood estimate are also provided, which are calculated directly from the samples using a method suitable for the particular algorithm used to calculate the marginal likelihood. As input, the inference tool requires three parts: a configuration file, an XML file specifying the prior, and a dynamic library that evaluates the likelihood function. For constructing the dynamic library that evaluates the likelihood function, BCM provides cross-platform boilerplate code, such that custom model simulation code can be easily adapted for use with BCM. Alternatively, the model parsing tool can be used as described further below.

The inference tool implements three classes of sampling algorithms: Markov chain Monte Carlo (MCMC) [6, 7], sequential Monte Carlo (SMC) [8] and nested sampling [9]. For each class of sampling algorithms, BCM includes several options for proposal distributions, as well as extensions that can increase the sampling efficiency when dealing with complex inference problems, giving a total of eleven different sampling algorithms (Table 1).

**Table 1:** Sampling algorithms and extensions implemented in BCM

Sampling algorithm	Reference
Markov Chain Monte Carlo	[6, 7]
Parallel tempering	[10]
Adaptive proposals	[11]
Feedback-optimized temperatures	[12]
Thermodynamic integration	[13]
Automated parameter blocking	[14]
Sequential Monte Carlo	[8]
MCMC proposals	[8]
Kernel density estimate proposals	[8]
Automated temperature schedule	[15]
Nested sampling	[9]
MCMC proposals	[9]
Ellipsoid proposals	[16]
MultiNest	[5]

Care has been taken to create efficient, multithreaded implementations of each algorithm. Firstly, the inference tool has been written in C++ and performance bottlenecks have been profiled and optimized. Secondly, each algorithm has been parallelized with a multithreading strategy suitable for that algorithm: for MCMC, multiple chains are distributed across threads, for SMC, particles are distributed in batches across threads, and for nested sampling, a batch of samples is generated at each iteration by all threads which are then re-used in subsequent nested sampling iterations.

The model parsing tool (*mdlparse*) is the second component of BCM. It can be used to generate the prior and likelihood files for the inference tool. The parsing tool reads a model description file that specifies the model, comprising the prior, likelihood and data references, and it outputs C++ source code for a dynamic library that evaluates the prior and likelihood function with the relevant data. This C++ code can then be used as a basis for further modification; or it can be directly compiled into a dynamic library. The input model description file uses a custom format with an easy-to-read syntax. An excerpt of a model description file is shown in Fig. 1b. The use of the model parsing tool is optional and it is meant as an aid in model specification rather than as a comprehensive tool capable of fully specifying all types of models.

Finally, a script is provided to load the output of the inference tool into R for further analysis and for visualization of the results. This script can be used to display kernel density estimates of the posterior probability distribution of the sampled variables, as well as to make plots for visual posterior predictive checking; examples of both of these are shown in Figs. 3 and 4. Basic functionality for convergence diagnostics is included as well, including autocorrelation functions and trace plots. Functions for conversion of the results to CODA objects [24] and to ggmcmc objects [25], two R packages for MCMC convergence diagnostics and output analysis, are also provided.

## Results

### Analytically tractable example

**Fig. 2:**
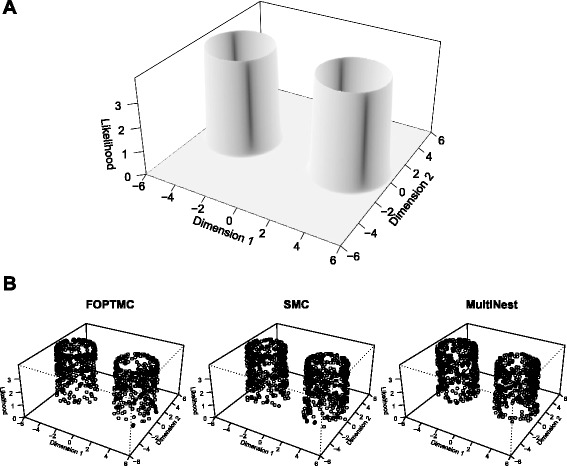
Gaussian shells example. **a** Likelihood of the Gaussian shells problem in the 2-dimensional case. **b** Samples generated from the likelihood by three sampling algorithms. In each case, the samples are well-distributed throughout each mode, and the two modes are sampled in approximately equal proportions

To showcase BCM, and to explore how each class of algorithms deals with increasing dimensionality and complex distributions, we first analyzed a problem which is analytically tractable: the Gaussian shells problem described in [5, 26]. While this example is not directly relevant for systems biology, its likelihood function is multimodal and ridge-shaped, resembling the likelihoods often encountered in systems biology models. The likelihood function for this Gaussian shells problem is given by

3 $$ P\left(\boldsymbol{\theta} \right)={\displaystyle \sum_{i=1}^2\frac{1}{\sqrt{2\pi {w}^2}} \exp \left(-\frac{{\left(\left|\boldsymbol{\theta} -{\boldsymbol{c}}_{\boldsymbol{i}}\right|-r\right)}^2}{2{w}^2}\right)} $$

where *r* = 2, *w* = 0.1, and ***Θ*** and ***c***_***i***_ are *n*-dimensional vectors. ***Θ*** is the vector of variables which are to be sampled and ***c***_***i***_ are two constant vectors describing the centers of the two peaks and are assigned the values *c*_1,*x*_ = 3.5, *c*_2,*x*_ = −3.5 and 0 in the other dimensions. This likelihood function is then composed of two narrow, well-separated, ring-shaped peaks (Fig. 2a), which is a challenging sampling problem.

We tested three sampling algorithms on this problem, one from each class of sampling algorithms: feedback-optimized parallel-tempered Markov chain Monte Carlo (FOPTMC) [12], sequential Monte Carlo (SMC) [8] with the automated temperature schedule selection of [15] but without using Approximate Bayesian Computation, and MultiNest [5].

As shown in Table 2, all three algorithms give the correct estimate for the marginal likelihood within the error bounds. When the number of dimensions is 10 or fewer, MultiNest is extremely efficient: it requires the fewest likelihood evaluations while achieving the tightest error bound. When the number of dimensions is increased beyond 10 however, MultiNest becomes very inefficient. At this point the exponential scaling of the algorithm becomes apparent. In the higher-dimensional setting, the SMC algorithm deals with this problem most efficiently. FOPTMC is least efficient: it requires the largest number of likelihood evaluations and has the largest error bound. FOPTMC can still effectively explore the posterior distribution (as shown in Fig. 2b), however, the temperature schedule of the parallel chains in FOPTMC is optimized for exploration of the posterior rather than for estimation of the marginal likelihood and as a result there is an increasingly large error in the marginal likelihood estimate at higher dimensionality.

**Table 2:** Performance of three sampling algorithms in calculating the marginal likelihood of an analytically tractable example

Dimensions	Log marginal likelihood				Likelihood evaluations (x1000)		
	Analytical	FOPTMC	SMC	MultiNest	FOPTMC	SMC	MultiNest
2	−1.75	−1.80 ± 0.68	−1.74 ± 0.39	−1.73 ± 0.29	147	79	18
5	−5.67	−5.98 ± 1.65	−5.66 ± 0.47	−5.73 ± 0.38	287	281	28
10	−14.59	−14.92 ± 3.34	−14.64 ± 0.62	−14.13 ± 0.63	969	521	95
30	−60.13	−61.11 ± 9.10	−59.85 ± 0.97	*	6420	1511	*
100	−255.62	−257.7 ± 24.8	−255.8 ± 1.54	*	96,251	4271	*

The following algorithms were used: *FOPTMC* feedback-optimized parallel-tempered Markov Chain Monte Carlo [12], *SMC* automated-temperature sequential Monte Carlo but without ABC approximation [15], and MultiNest [5]. The column ‘Analytical’ gives the marginal likelihood value calculated analytically. (*) indicates that the computation time exceeded the maximal time of 1 h; the other calculations required at most 5 min

### Kinetic ordinary differential equation model

**Fig. 3:**
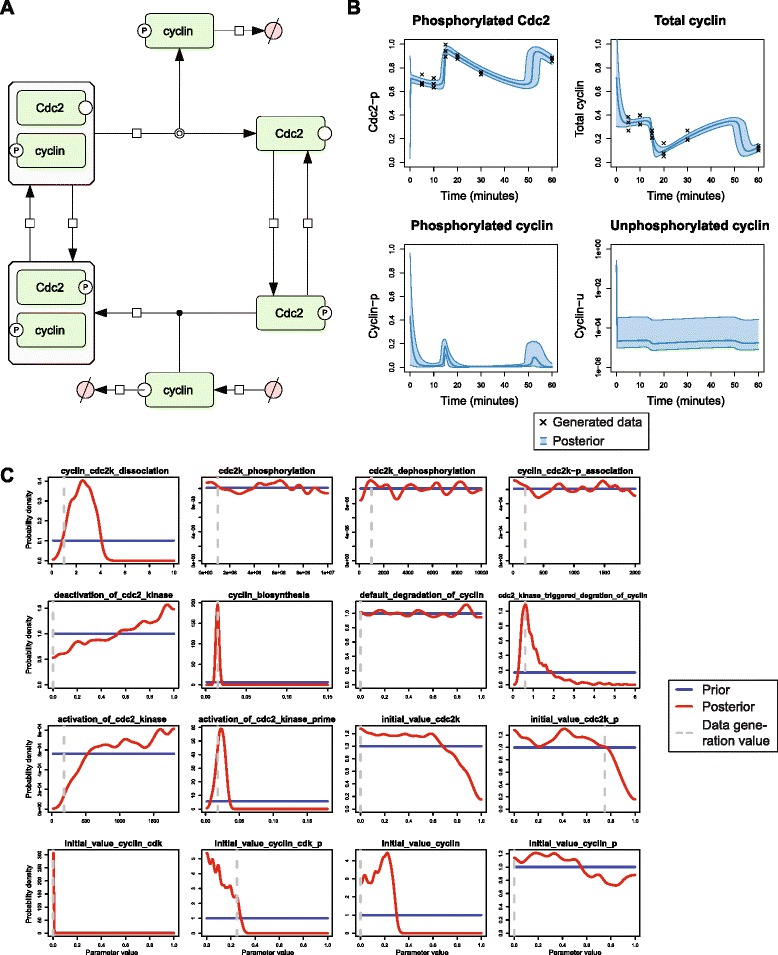
Analysis of an ODE-based model of the cell cycle. **a** Graphical representation of the cell cycle model of Tyson [27]. **b** Posterior distribution of the two observables; phosphorylated Cdc2 and the total amount of cyclin, and of two unobserved species, phosphorylated and unphosphorylated cyclin. The black crosses represent the generated data which are used for the inference. The shaded blue area represents the posterior 95 % confidence interval of the mean of the observables. **c** The prior and posterior probability distributions of each of the 16 parameters. The blue lines indicate the prior, the red lines the estimated posterior, and the dashed grey lines indicate the values that were used to generate the data. The densities are estimated from the posterior samples using kernel density estimation with Sheather-Jones bandwidth selection

Having explored the behavior of several sampling algorithms in an analytically tractable example, we now illustrate the use of BCM for analyzing biological computational models. As an example of this, we investigated the inference of the parameters of a model based on a system of ordinary differential equations (ODEs). The 6-variable cell cycle model of Tyson [27] was used, as downloaded from BioModels [17]. A graphical representation of this model is shown in Fig. 3a.

To recreate a typical setting in biology, data was generated from the model at six time points for two observables with three replicates (see Additional file 1). Then BCM was used to infer all 16 parameters of the model (10 kinetic parameters and 6 initial conditions) from these 36 data points. The priors for the kinetic parameters were set to a uniform distribution spanning an order of magnitude on either side of the parameter values that were used to generate the data, and the priors for the initial conditions were set to a uniform distribution between 0 and 1 (see blue curves in Fig. 3c). The likelihood function was set equal to the one that generated the data, that is, a normal distribution with standard deviation 0.05.

Despite the small size of the model, this inference problem is challenging. Firstly, the ODE system is stiff, and even with the use of an implicit ODE solver it is costly to simulate. Secondly, there are multiple distinct ways in which the model can fit the data, leading to sub-optimal modes in the posterior distribution. Thus, a sampler must be able to escape these local optima, and it must be able to converge to the correct posterior distribution with a limited number of likelihood evaluations due to the computational cost of the simulations.

Four sampling algorithms were tested on this problem: SMC, MultiNest, FOPTMC (now extended with automated parameter blocking [14]), and additionally nested sampling with MCMC proposals (Nested-MCMC) was added as an alternative nested sampling strategy. In this inference task, FOPTMC with automated parameter blocking was most efficient, requiring 14 h to generate 2000 samples from the posterior. SMC required 19 h, while Nested-MCMC required 30 h and MultiNest had to be discontinued as the acceptance rate quickly dropped to essentially zero. The tests were performed using 16 threads on an Intel Xeon E5-2680 processor.

The Bayesian estimates of the parameters and the trajectories of the species can be used to study the uncertainty in the model. Figure 3b shows the posterior distribution of the two observables, as well as of two inferred species for which no observable data was generated, as estimated by FOPTMC. We can see that the data are sufficient to constrain the trajectories of the observed species. For the unobserved species phosphorylated cyclin, the overall trajectory can also be inferred. Nevertheless, for this unobserved species, the second peak is more variable – here the data is insufficient to constrain the precise magnitude of the peak. For the other unobserved species, unphosphorylated cyclin, we see that there is greater uncertainty. The posterior distribution indicates only that the average levels are low, but the precise levels cannot be inferred from these data.

Figure 3c shows the marginal posterior probability distributions of the parameters. It can be seen that for all parameters, the values used to generate the data fall within areas of non-zero probability of the posterior. In most cases the data-generation values also have maximum posterior probability, but interestingly this is not true for all parameters, such as for the activation and deactivation of Cdc2. Furthermore, some parameters are not identifiable, for example the rates of phosphorylation and desphosphorylation of Cdc2 cannot be determined from the data. In general, such lack of identifiability could be for structural reasons, that is, the parameters cannot be inferred in theory given the observed species, due to a redundant parameterization. Alternatively, the parameters may be identifiable in theory, but the data may provide insufficient information to constrain the parameters in practice.

Overall, the Bayesian estimates provide useful measures of the uncertainty in parameter values, model fit and model predictions.

### Comparison with existing software packages

There are several software packages which can perform Bayesian inference of the parameters of ODE-based models: BioBayes [21], ABC-SysBio [22], SYSBIONS [23] and Stan [20]. BioBayes uses parallel-tempered Markov Chain Monte Carlo, ABC-SysBio uses sequential Monte Carlo sampling in combination with Approximate Bayesian Computation, SYSBIONS uses nested sampling, and Stan uses Hamiltonian Monte Carlo and the No-U-Turn sampler (NUTS).

**Fig. 4:**
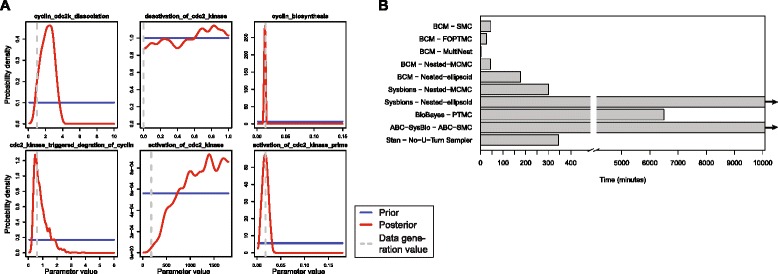
Performance comparison of BCM with existing software packages. **a** Prior and posterior probability distributions of the 6 parameters of the simplified inference problem. **b** Time required for generating 1000 samples from the posterior using BCM, SYSBIONS, BioBayes, ABC-SysBio and Stan, with several different sampling algorithm. The sampling was terminated if it had not converged after 7 days

To compare BCM with these software packages, a simplified version of the previous inference problem was used. Instead of inferring all 16 parameters, the initial conditions and 4 of the 10 kinetic parameters were fixed to the values used to generate the data, leaving 6 parameters to be inferred. Figure 4a shows the marginal posterior probability distributions of the simplified problem, as estimated by BCM using FOPTMC (see Additional file 2: Figure S1 for the posteriors estimated by each algorithm/software package). The other software packages were optimized for this problem as much as possible to give a fair comparison (see Additional file 1).

Figure 4b shows the time required to generate 1000 samples from the posterior with each software package and algorithm, using eight threads on an Intel Xeon E5-2680 processor. It is clear that BCM is significantly faster than the other software packages. In particular the MultiNest algorithm in BCM is extremely efficient in this low-dimensional setting, requiring only 75 s. The other algorithms in BCM required between 25 and 50 min, except for ellipsoidal nested sampling which required three hours. From the other software packages, only SYSBIONS and Stan were able to solve this inference problem in a reasonable amount of time. SYSBIONS required five hours using Nested-MCMC, which is approximately six times longer than BCM with the same algorithm. For Stan, using the NUTS algorithm, the sampling with a chain does not always converge as the NUTS algorithm does not have a means to escape sub-optimal modes. This problem was addressed by starting eight separate chains in parallel, in which case most of the chains were sampling the correct, optimal mode. In this case, Stan required approximately six hours to generate the requested samples. BioBayes was able to reach apparent convergence in 4.5 days. For ABC-SysBio, and SYSBIONS using ellipsoidal sampling, the samplers did not reach convergence in 7 days (see Additional file 1).

## Conclusion

The BCM toolkit provides efficient, multithreaded implementations of eleven sampling algorithms for generating posterior samples and calculating marginal likelihoods. Additional tools are included which facilitate the process of specifying models and visualizing the sampling output. This toolkit can be used for analyzing the uncertainty in the parameters and the predictions of computational models using Bayesian statistics.

The examples show that it depends on the problem which sampling algorithm will perform well. In the Gaussian shells example, where the focus was on marginal likelihood estimation, MultiNest performed best in a low-dimensional setting, and in the medium- to high dimensional setting sequential Monte Carlo was most efficient. In the cell cycle example, where the focus was on parameter inference, parallel-tempered Markov chain Monte Carlo was more efficient than sequential Monte Carlo. There are various aspects of the posterior probability distribution which affect the performance of the different algorithms; for example the number of modes, how well the shapes of the modes are approximated by the proposal distributions, and the location and volume of the posterior modes with respect to the prior. These features of the posterior probability distribution will typically not be known for the problem of interest before starting the analysis, and it is then unclear which algorithm might be most suitable. The availability of various algorithms in BCM will therefore be useful in the Bayesian analysis of diverse models.

In the second example, we have shown that BCM can be used to infer the parameters of an ODE-based model of the cell cycle. BCM is significantly more efficient in this task than existing software packages. This increase in efficiency was possible due to the parallelization of the sampling algorithms in combination with the use of optimized C++ as programming language. Due to the higher efficiency, BCM allows the analysis of larger or more challenging computational models than was previously feasible. In previous cases where Bayesian analysis of complex biological computational models was done, such as in [3, 4, 28], sampling algorithms were newly implemented for each project. The availability of BCM as an efficient, reusable software package can help in streamlining such projects in the future.

## Availability and requirements

**Project name**: BCM – toolkit for Bayesian analysis of Computational Models using samplers

**Project home page**: http://ccb.nki.nl/software/bcm/

**Operating systems**: Windows, Linux, Mac

**Programming language**: C++ and R

**Dependencies**: Boost C++ libraries (tested with version 1.55.0), CMake (version 3.2 or later).

**License**: Mozilla Public License 2.0

## Additional files

Additional file 1: Supplementary Information. Description: Supplementary information describing the methodological details and all settings that were used for each inference. (DOCX 49 kb)
Additional file 2: Figure S1. Description: Overview of the sampling results of each inference of the comparison with existing software packages. (PDF 1 mb)

## Abbreviations

ABC: Approximate Bayesian Computation
BCM: Toolkit for Bayesian analysis of Computational Models using samplers
FOPTMC: Feedback-optimized parallel-tempered Markov chain Monte Carlo
MCMC: Markov chain Monte Carlo
NUTS: No-U-turn sampler
SMC: Sequential Monte Carlo

## References

[1] Wilkinson DJ (2007). Bayesian methods in bioinformatics and computational systems biology. Brief Bioinform.

[2] Vyshemirsky V, Girolami M (2008). Bayesian ranking of biochemical system models. Bioinformatics.

[3] Xu T-R, Vyshemirsky V, Gormand A, von Kriegsheim A, Girolami M, Baillie GS, Ketley D, Dunlop AJ, Milligan G, Houslay MD, Kolch W (2010). Inferring signaling pathway topologies from multiple perturbation measurements of specific biochemical species. Sci Signal.

[4] Eydgahi H, Chen WW, Muhlich JL, Vitkup D, Tsitsiklis JN, Sorger PK (2013). Properties of cell death models calibrated and compared using Bayesian approaches. Mol Syst Biol.

[5] Feroz F, Hobson MP, Bridges M (2009). MultiNest: an efficient and robust Bayesian inference tool for cosmology and particle physics. Mon Not R Astron Soc.

[6] Metropolis N, Rosenbluth AW, Rosenbluth MN, Teller AH (1953). Equation of state calculations by fast computing machines. J Chem Phys.

[7] Hastings WK (1970). Monte Carlo sampling methods using Markov chains and their applications. Biometrika.

[8] Del Moral P, Doucet A, Jasra A (2006). Sequential Monte Carlo samplers. J R Stat Soc Ser B (Stat Methodol).

[9] Skilling J (2006). Nested sampling for general Bayesian computation. Bayesian Anal.

[10] Geyer CJ (1991). Markov chain Monte Carlo maximum likelihood. Proceedings of the 23rd symposium interface.

[11] Haario H, Saksman E, Tamminen J (2001). An adaptive metropolis algorithm. Bernoulli.

[12] Katzgraber HG, Trebst S, Huse DA, Troyer M. Feedback-optimized parallel tempering Monte Carlo. J Stat Mech Theory Exp. 2006. doi:10.1088/1742-5468/2006/03/P03018.

[13] Gelman A, Meng X-L (1998). Simulating normalizing constants: from importance sampling to bridge sampling to path sampling. Stat Sci.

[14] Turek D, de Valpine P, Paciorek CJ, Anderson-Bergman C. Automated parameter blocking for efficient Markov chain Monte Carlo sampling. Bayesian Anal. 2016. in press.

[15] Del Moral P, Doucet A, Jasra A (2011). An adaptive sequential Monte Carlo method for approximate Bayesian computation. Stat Comput.

[16] Mukherjee P, Parkinson D, Liddle AR (2006). A nested sampling algorithm for cosmological model selection. Astrophys J.

[17] Chelliah V, Juty N, Ajmera I, Ali R, Dumousseau M, Glont M, Hucka M, Jalowicki G, Keating S, Knight-Schrijver V, Lloret-Villas A, Natarajan KN, Pettit JB, Rodriguez N, Schubert M, Wimalaratne SM, Zhao Y, Hermjakob H, Le Novère N, Laibe C (2015). BioModels: ten-year anniversary. Nucleic Acids Res.

[18] Girolami M (2008). Bayesian inference for differential equations. Theor Comput Sci.

[19] Lunn D, Spiegelhalter D, Thomas A, Best N (2009). The BUGS project: evolution, critique and future directions. Stat Med.

[20] Carpenter B, Gelman A, Hoffman M, Lee D, Goodrich B, Betancourt M, Brubaker MA, Guo J, Li P, Riddell A. Stan: a probabilistic programming language. J Stat Softw. 2015. in press. 10.18637/jss.v076.i01 PMC9788645 36568334

[21] Vyshemirsky V, Girolami M (2008). BioBayes: a software package for Bayesian inference in systems biology. Bioinformatics.

[22] Liepe J, Barnes C, Cule E, Erguler K, Kirk P, Toni T, Stumpf MPH (2010). ABC-SysBio—approximate bayesian computation in python with GPU support. Bioinformatics.

[23] Johnson R, Kirk P, Stumpf MPH (2014). SYSBIONS: nested sampling for systems biology. Bioinformatics.

[24] Plummer M, Best N, Cowles K, Vines K (2006). CODA: Convergence Diagnosis and Output Analysis for MCMC. R News.

[25] Fernández-i-Marín X. ggmcmc: analysis of MCMC samples and Bayesian inference. J Stat Softw. 2016;70(9):1–20.

[26] Allanach BC, Lester CG (2008). Sampling using a “bank” of clues. Comput Phys Commun.

[27] Tyson JJ (1991). Modeling the cell division cycle: cdc2 and cyclin interactions. Proc Natl Acad Sci U S A.

[28] Milias-Argeitis A, Oliveira AP, Gerosa L, Falter L, Sauer U, Lygeros J (2016). Elucidation of genetic interactions in the yeast GATA-factor network using Bayesian model selection. PLoS Comput Biol.

