# Supplementary material for: BCM: toolkit for Bayesian analysis of Computational Models using samplers
Source: BMC Syst Biol. 2016 Oct 21;10:100. doi: 10.1186/s12918-016-0339-3 (PMC5073811; doi:10.1186/s12918-016-0339-3)
Supplement: Additional file 1: — Supplementary Information. Description: Supplementary information describing the methodological details and all settings that were used for each inference. (DOCX 49 kb) [file 12918_2016_339_MOESM1_ESM.docx]

Supplementary Information

# Supplementary Methods – Gaussian Shells example

To sample from the Gaussian Shells likelihood, three different samplers were used. Each sampler is briefly introduced below, along with the specific settings that were used.

#### Feedback-optimized parallel-tempered Markov chain Monte Carlo

The algorithm described in [1] was used. Briefly, a number of parallel Markov chain Monte Carlo chains are constructed, where each chain samples from a power posterior given by the equation

$$P_{\beta}(\theta)\propto P(X|\theta)^{\beta}P(\theta)$$

and the temperature parameter *β* varies between the chains. A temperature of 0 corresponds to sampling from the prior and a temperature of 1 corresponds to sampling from the posterior. At each sampling iteration, a choice is made at random between either updating all the chains separately with a Metropolis-Hastings step, or swapping two neighboring chains with probability

$$\min(\frac{P_{\beta_{i}}(\Theta_{j})P_{\beta_{j}}(\Theta_{i})}{P_{\beta_{i}}(\Theta_{i})P_{\beta_{j}}(\Theta_{j})},1)$$

where *i* and *j* are the indices of the neighboring chains.

The feedback-optimization refers to optimizing the temperatures of the parallel chains such that there is a minimal round-trip time between the prior and the posterior. That is, the number of swaps required for a sample to diffuse from the posterior to the prior and back is minimized by choosing a particular set of temperatures according to the feedback-optimization algorithm.

The settings for FOPTMC were as follows. First, two iterations of temperature optimization were run, with 200 sampling iterations each, and a new temperature set was recalculated afterwards. Following these two iterations, the posterior distribution was sampled for 2,000 iterations. No additional burn-in time was used; the temperature optimization steps served as burn-in. The subsampling during the temperature optimizations and during the final sampling period was adjusted for the dimensionality as given in Supplementary Table 1. These choices of subsampling were made such that there were at least 100 round-trips from prior to posterior in the final sampling stage. The number of parallel chains was set to 16 for the 2-dimensional problem and increased for higher dimensions, as given in Supplementary Table 1. These numbers were chosen such that the average acceptance rate of the chain swaps was at least 20% for any pair of chains after temperature optimization.

The marginal likelihood was calculated with thermodynamic integration [2] with a trapezoidal integration rule.

Supplementary Table 1: Number of chains and subsampling for the parallel-tempered Markov chain Monte Carlo sampler.

| **Dimensions** | **Chains** | **Subsampling** |
| --- | --- | --- |
| 2 | 16 | 1 in 250 |
| 5 | 16 | 1 in 500 |
| 10 | 24 | 1 in 1,000 |
| 30 | 32 | 1 in 5,000 |
| 100 | 48 | 1 in 50,000 |

#### Sequential Monte Carlo

The sequential Monte Carlo algorithm described in [3] was used, complemented with the automated temperature selection described in [4], but without using Approximate Bayesian Computation (ABC). The ABC approximation is not necessary since the likelihood function can be evaluated in closed-form.

Briefly, in sequential Monte Carlo sampling, a sequence of distributions is constructed between the prior and posterior. A collection of samples is first generated from the prior and these are propagated sequentially between the distributions. The same power posterior is used as for the FOPTMC algorithm:

$$P_{\beta}(\theta)\propto P(X|\theta)^{\beta}P(\theta)$$

The temperature *β* of the next iteration is calculated using the expected effective sample size (ESS) of the next iteration:

$$\mathrm{ESS}=1/\sum_{i=1}^{N} (W_{n}^{i})^{2}$$

where $W_{n}^{i}$ is the weight of the *i*th sample in the *n*th distribution. The ESS of the next iteration, which depends on the temperature of that iteration, cannot be calculated exactly but can be approximated using the weights from the current distribution. Using this expected ESS, the algorithm searches for a temperature *β_n_* such that the expected ESS of the next iteration is at least α times the current ESS, where the parameter α can be specified between 0 and 1. This parameter α controls how much the iterations are allowed to differ from each other. When the ESS drops below a certain threshold, the particles are resampled so that the samples with high weight are re-used and the low-weight particles are discarded.

The settings for SMC were as follows. A population of 1,000 samples was used and α was set to 0.9. The ESS-threshold for resampling was set to half the population size, and the resampling was done using residual resampling. For sample updating, a Metropolis-Hastings kernel was used, with a Gaussian proposal distribution with diagonal covariance matrix. The variances were set to the empirical variances of the previous population of samples, and scaled such that the average acceptance rate was 0.22. In each SMC iteration, 10 Metropolis-Hastings steps were taken.

The marginal likelihood was calculated using the weights at each resampling step as described in section 3.2.1 of [3].

#### MultiNest

The MultiNest algorithm described in [5] was used. Briefly, in nested sampling [6], a collection of samples is first generated from the prior. Next, in each iteration, the sample with the lowest likelihood is discarded, and a new sample is generated with likelihood strictly higher than the one that was discarded. In MultiNest, the proposal distribution for generating new samples is a collection of ellipsoids that is fitted to the current population of samples. This collection of ellipsoids is constructed using a combination of K-means clustering and progressive updating, to achieve a collection of ellipsoids with minimal total volume while still bounding all the current samples.

The settings for MultiNest were as follows. A population of 1,000 samples was used, and the enlargement factor of the ellipsoids was set to 1.1. The collection of ellipsoids was updated every 250 steps; for the steps in between ellipsoid recalculations, only the scaling of the ellipsoids was adjusted as necessary to maintain bounds. The sampling was terminated when the number of steps taken was greater than the current information estimate times the population size, multiplied by a safety factor (this is the original nested sampling termination criterion from [6]). The safety factor was set to 1.2.

# Supplementary Methods – Cell cycle example with 16 parameters

### Data generation

The 6-variable cell cycle model of Tyson [7] was used and downloaded from BioModels [8] with ID BIOMD0000000005. The model was simulated with the parameter values and initial conditions supplied with the model using COPASI [9], using LSODA as ODE integrator, with very small error tolerance (10^-9^ relative, 10^-12^ absolute). Then, for 6 time points (5, 10, 15, 20, 30 and 60 minutes), 3 data points each were generated by adding normally distributed noise with a standard deviation of 0.05. This was done for two observables: the species p-Cdc2, and the total amount of cyclin. The total amount of cyclin is a sum of 4 species in the model.

### Parameter inference

Four different samplers were used to infer the parameters of the model from the generated data: FOPTMC, SMC, MultiNest, and Nested-MCMC. The first three were also used in the Gaussian shells example. Nested-MCMC is run in the same manner as MultiNest, except that an MCMC proposal distribution is used instead of the MultiNest-proposals. The specific settings for each sampling algorithm are listed below.

During the inference, the ODE system is integrated with CVODE [10], with relative and absolute error tolerance of 10^-6^.

#### Feedback-optimized parallel-tempered Markov chain Monte Carlo

The number of parallel chains was set to 32. Two rounds of temperature optimization with 1,000 samples each were run, followed by an additional burn-in period of 1,000 samples and a final sampling stage of 2,000 samples. A subsampling of 1 in 2,500 was used. One sample (before subsampling) consists of updating each parameter once. Proposals were adapted every 500 samples (after subsampling).

Additionally, the automated parameter blocking of [11] was used. Briefly, this method works as follows. Initially a scalar Gaussian proposal distribution is used for each parameter separately. Then at each proposal-update-step, the empirical correlation between all parameters over the last period is calculated. This correlation matrix is clustered using hierarchical clustering with complete linkage. The hierarchical tree is then cut at a correlation of 0.5. In the next sampling period, the groups of parameters which are clustered together are sampled together using a Gaussian proposal distribution with full covariance matrix based on the empirical covariance of the samples. Parameters which are not correlated with any of the other parameters continue to use the scalar Gaussian proposal distribution. The scale of each proposal distribution was continuously adapted to maintain an average acceptance rate of 0.22, as calculated using an exponential moving average with a period of 1/10^th^ of the proposal update interval.

#### Sequential Monte Carlo

The population size was set to 4,096, and the temperature selection parameter α was set to 0.999. A Gaussian proposal kernel with diagonal covariance matrix was used, and scaled such that the average acceptance rate was 0.22. In each SMC iteration, 20 Metropolis-Hastings steps were taken for each particle.

#### MultiNest

A population of 2,000 samples was used, and the enlargement factor of the ellipsoids was set to 1.1. The collection of ellipsoids was updated every 500 steps. The sampling did not reach the termination criterion – after approximately 6,000 steps at an information estimate of 8.05, the acceptance rate dropped to essentially 0 and the sampling was terminated as no additional steps were being made.

#### Nested sampling with Markov chain Monte Carlo proposals

A population of 2,000 samples was used. For the generation of new samples, a random sample from the current population was chosen, which was then updated using 5,000 Metropolis-Hastings steps. The proposal distribution was a Gaussian kernel with diagonal covariance matrix, and scaled such that the average acceptance rate was 0.22.

# Supplementary Methods – Cell cycle example with 6 parameters

To simplify the inference problem, all 6 initial conditions and 4 of the 10 kinetic parameters were fixed to the values used to generate the data. The 6 remaining parameters were chosen to include both identifiable and unidentifiable parameters, and to still give rise to sub-optimal models in the posterior probability distribution.

The inference in BCM was run in the same way as was done for the full 16-parameter inference problem, with the following modifications:

*FOPTMC*: Eight parallel chains, and a subsampling of 1 in 500.
*SMC*: Population size of 2,048, temperate selection α of 0.99 and 10 MCMC steps per iteration.
*MultiNest*: Population size of 1,000.
*Nested-MCMC*: Population size of 1,000 and 500 MCMC steps per iteration.
*Nested-Ellipsoid*: Population size of 1,000 and an ellipsoid enlargement of 1.1.

For the inference in SYSBIONS, the algorithm settings were set identical to the corresponding algorithm in BCM, as listed below. The exception is that SYSBIONS does not support the termination criterion based on the information estimate; instead SYSBIONS was run with increasing number of iterations until 1,000 posterior samples were generated. We used SYSBIONS version 03.

*Nested-MCMC*: 1,000 live points and 500 MCMC steps per iteration.
*Nested-ellipsoid*: 1,000 live points and an ellipsoid enlargement of 1.1.

For the inference in ABC-SysBio, the target epsilon was set to 0.2. This target epsilon should be feasible given that the data standard deviation of the data is 0.05. Both an automated and a fixed schedule were run; the automated schedule with α=0.9, and the fixed schedule as follows: [100.0 2.0 1.0 0.9 0.8 0.7 0.6 0.5 0.45 0.40 0.35 0.30 0.25 0.20]. However, neither of the two epsilon schedules managed to reach the final epsilon value in 7 days. We used ABC-SysBio version 2.08.

For the inference in BioBayes, the Population MCMC algorithm was used, with 8 parallel chains, a burn-in period of 100 and posterior sample size of 1,000. Sample thinning was set to 1 in 50,000. Convergence monitoring was not used. We used BioBayes version 1.3. Note that the inferred posterior probabilities are different in BioBayes compared to the other inference programs; we believe this may be due to different tolerance settings in the ODE integrator.

For the inference in Stan, the No-U-Turn sampler (NUTS) was used. The sampler was run for 500 iterations, with 250 warmup/adaptation iterations, and 250 sampling iterations. All control parameters were left at their default setting. The ODE integration tolerances were set to 10^-6^, and the maximum number of solver iterations to 500, so that these settings were the same as those that were used in BCM, SYSBIONS and ABC-SysBio. We used 8 parallel chains. Note that this provides more than the required samples, however the chains are sampling in parallel using the 8 available threads and so do not increase the computation time. The eight chains are also required to have sufficient chance of at least some of the chains to sample from the optimal mode. We used Stan version 2.11.

# Supplementary Methods – Software package optimization

Several modifications were made to the other software packages to optimize them for the parameter inference of the Tyson cell cycle model.

SYSBIONS uses the Dormand-Prince method for solving ODE systems [12], but this method is inefficient for simulating the present model. The ODE solver was therefore replaced with CVODE [10], significantly speeding up the inference. Note that CVODE was also used in the model implementation in BCM, and the same algorithm is used by ABC-SysBio and Stan.

For ABC-SysBio, we tested both the Python and C++ versions, and both small and large time steps for the ODE solver. We found that in particular larger time steps (of 1 minute) significantly sped up the evaluation of the likelihood function. However, none of the settings managed to reach convergence within 7 days, due to both slow simulation of the model (approximately 150 ms in ABC-SysBio, compared to approximately 5 ms in SYSBIONS or BCM) and low acceptance rates.

For BioBayes, the model was modified slightly for it to be able to load into the program. Specifically, the assignment rules, function specifications and annotations were removed; these modifications do not affect the dynamics of the species in the model.

# References

1. Katzgraber HG, Trebst S, Huse DA, Troyer M: **Feedback-optimized parallel tempering Monte Carlo**. *J Stat Mech Theory Exp* 2006, **2006**:P03018.

2. Gelman A, Meng X-L: **Simulating normalizing constants: from importance sampling to bridge sampling to path sampling**. *Stat Sci* 1998, **13**:163–185.

3. Del Moral P, Doucet A, Jasra A: **Sequential Monte Carlo samplers**. *J R Stat Soc Ser B (Statistical Methodol* 2006, **68**:411–436.

4. Del Moral P, Doucet A, Jasra A: **An adaptive sequential Monte Carlo method for approximate Bayesian computation**. *Stat Comput* 2011, **22**:1009–1020.

5. Feroz F, Hobson MP, Bridges M: **MultiNest: an efficient and robust Bayesian inference tool for cosmology and particle physics**. *Mon Not R Astron Soc* 2009, **398**:1601–1614.

6. Skilling J: **Nested sampling for general Bayesian computation**. *Bayesian Anal* 2006, **1**:833–859.

7. Tyson JJ: **Modeling the cell division cycle: cdc2 and cyclin interactions.** *Proc Natl Acad Sci U S A* 1991, **88**:7328–7332.

8. Chelliah V, Juty N, Ajmera I, Ali R, Dumousseau M, Glont M, Hucka M, Jalowicki G, Keating S, Knight-Schrijver V, Lloret-Villas A, Natarajan KN, Pettit JB, Rodriguez N, Schubert M, Wimalaratne SM, Zhao Y, Hermjakob H, Le Novère N, Laibe C: **BioModels: Ten-year anniversary**. *Nucleic Acids Res* 2015, **43**:D542–D548.

9. Hoops S, Gauges R, Lee C, Pahle J, Simus N, Singhal M, Xu L, Mendes P, Kummer U: **COPASI - A COmplex PAthway SImulator**. *Bioinformatics* 2006, **22**:3067–3074.

10. Cohen SD, Hindmarsh AC: **CVODE, A Stiff/Nonstiff ODE Solver in C**. *Comput Phys* 1996, **10**:138–143.

11. Turek D, de Valpine P, Paciorek CJ, Anderson-Bergman C: **Automated Parameter Blocking for Efficient Markov-Chain Monte Carlo Sampling**. *arXiv Prepr* 2015:1503.05621.

12. Dormand JR, Prince PJ: **A family of embedded Runge-Kutta formulae**. *J Comput Appl Math* 1980, **6**:19–26.
