# Supplementary figures and images for: BCM: toolkit for Bayesian analysis of Computational Models using samplers
Source: BMC Syst Biol. 2016 Oct 21;10:100. doi: 10.1186/s12918-016-0339-3 (PMC5073811; doi:10.1186/s12918-016-0339-3)

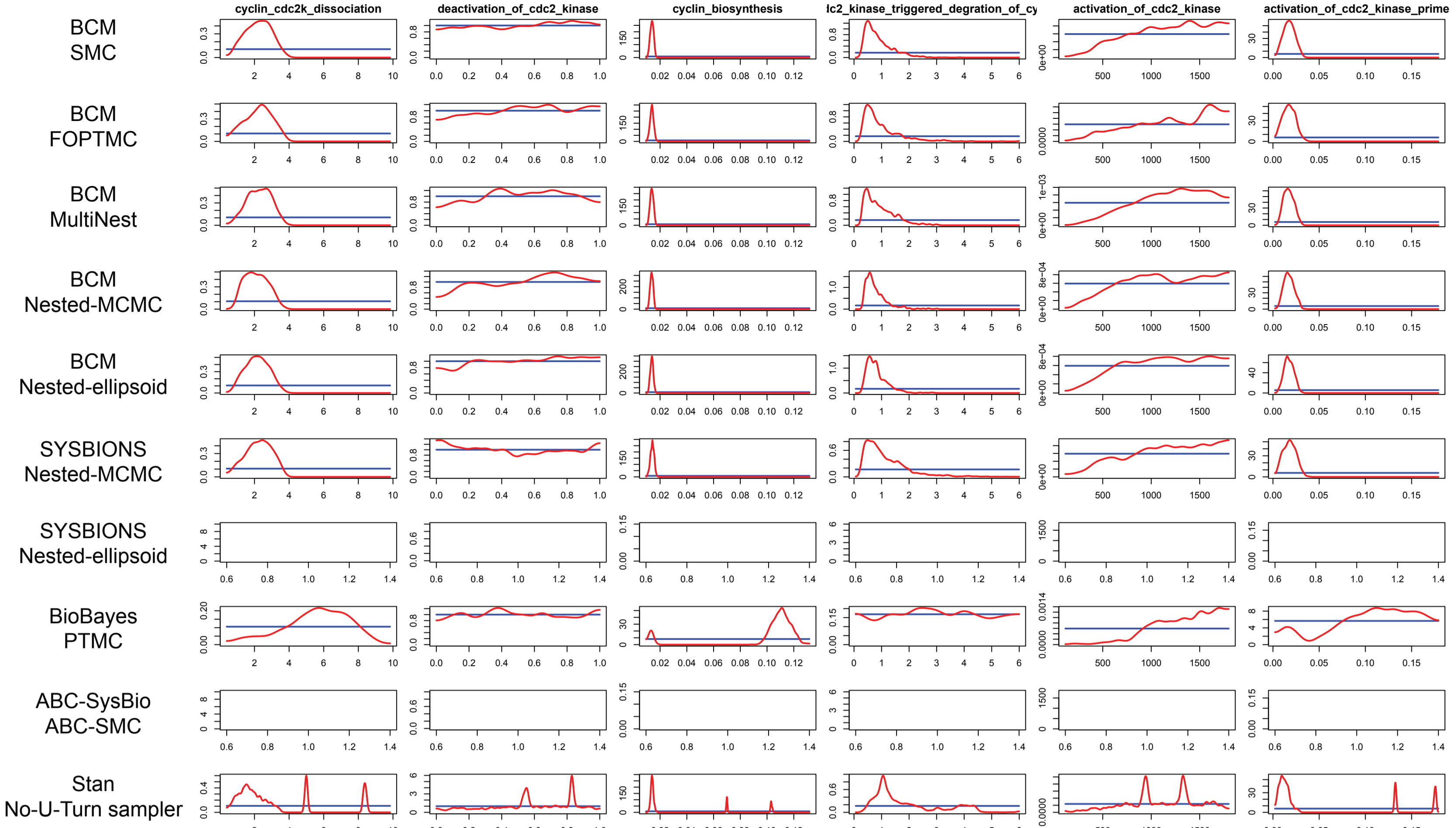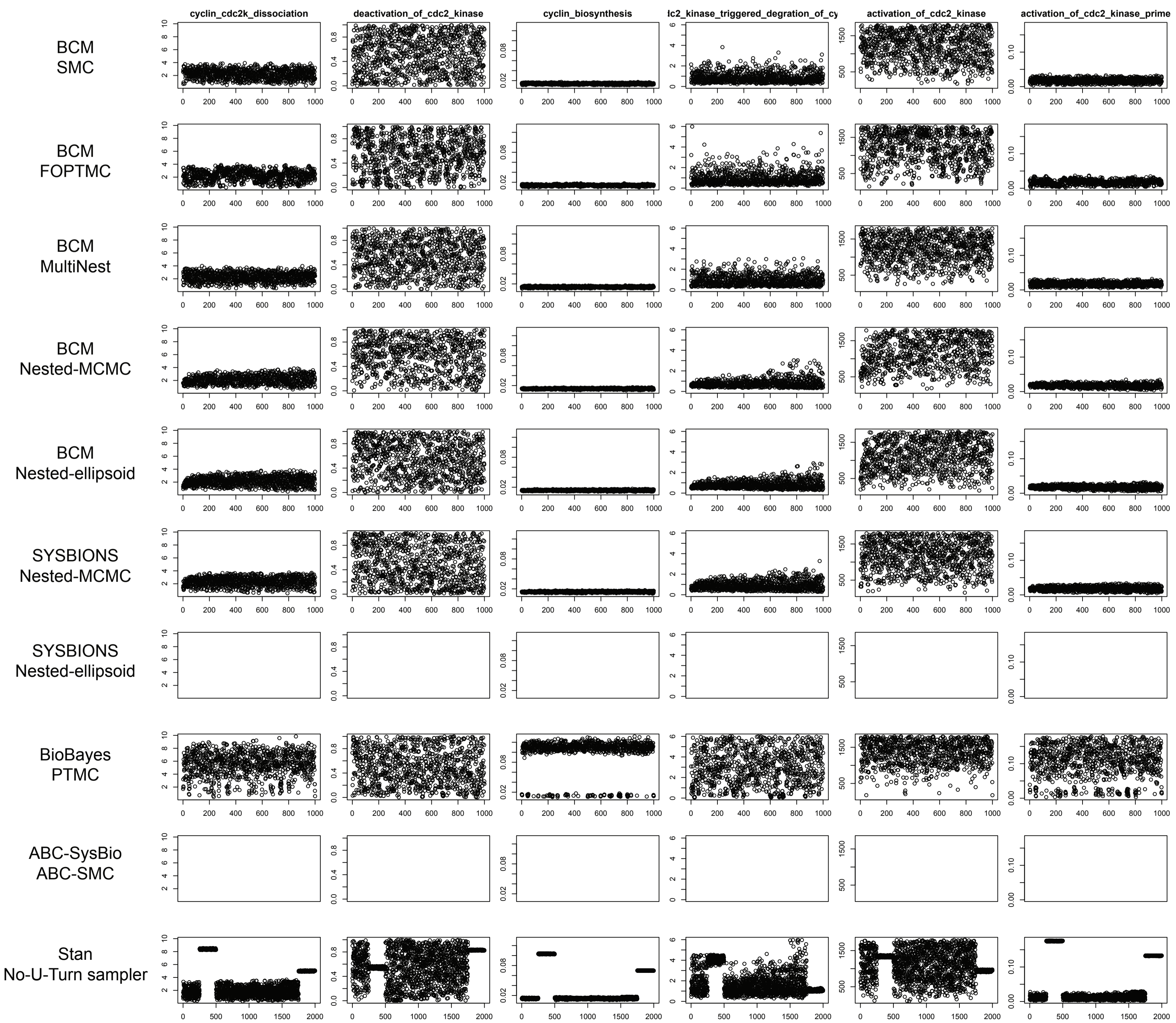

Supplement: Additional file 2: Figure S1. — Description: Overview of the sampling results of each inference of the comparison with existing software packages. (PDF 1 mb) [file 12918_2016_339_MOESM2_ESM.pdf]
